# Multiplex LC-MS/MS Assays for Clinical Bioanalysis of MEDI4276, an Antibody-Drug Conjugate of Tubulysin Analogue Attached via Cleavable Linker to a Biparatopic Humanized Antibody against HER-2

**DOI:** 10.3390/antib8010011

**Published:** 2019-01-11

**Authors:** Morse Faria, Marlking Peay, Brandon Lam, Eric Ma, Moucun Yuan, Michael Waldron, William R. Mylott, Meina Liang, Anton I. Rosenbaum

**Affiliations:** 1 PPD Laboratories, 2244 Dabney Road, Richmond, VA 23230, USA; Marlking.Peay@ppdi.com (M.P.); Eric.Ma@ppdi.com (E.M.); Moucun.Yuan@ppdi.com (M.Y.); Michael.Waldron@ppdi.com (M.W.); William.Mylott@ppdi.com (W.R.M.Jr.); 2 Clinical Immunology and Bioanalysis, Clinical Pharmacology and DMPK, MedImmune LLC, 121 Oyster Point Boulevard, South San Francisco, CA 94080, USA; blam@cytomx.com (B.L.); LiangM@MedImmune.com (M.L.); 3 Present address: Pharmacology & Toxicology, CytomX Therapeutics, Inc., 151 Oyster Point Boulevard, Suite 400, South San Francisco, CA 94080, USA

**Keywords:** Antibody-drug conjugates (ADCs), Hybrid LBA-LC-MS/MS, Multiplexing, Tubulysin, Total ADC, Total Antibody, unconjugated payload, MEDI4276, AZ13599185, AZ13687308

## Abstract

Bioanalysis of complex biotherapeutics, such as antibody-drug conjugates (ADCs), is challenging and requires multiple assays to describe their pharmacokinetic (PK) profiles. To enable exposure-safety and exposure-efficacy analyses, as well as to understand the metabolism of ADC therapeutics, three bioanalytical methods are typically employed: Total Antibody, Antibody Conjugated Toxin or Total ADC and Unconjugated Toxin. MEDI4276 is an ADC comprised of biparatopic humanized antibody attached via a protease-cleavable peptide-based maleimidocaproyl linker to a tubulysin toxin (AZ13599185) with an approximate average drug-antibody ratio of 4. The conjugated payload of MEDI4276 can undergo ester hydrolysis to produce the conjugated payload AZ13687308, leading to the formation of MEDI1498 (de-acetylated MEDI4276). In this report, we describe the development, validation and application of three novel multiplex bioanalytical methods. The first ligand-binding liquid chromatography coupled with tandem mass spectrometry (LBA-LC-MS/MS) method was developed and validated for simultaneous measurement of total antibody and total ADC (antibody-conjugated AZ13599185) from MEDI4276. The second LBA-LC-MS/MS assay quantified total ADC (antibody-conjugated AZ13687308) from MEDI1498. The third multiplex LC-MS/MS assay was used for simultaneous quantification of unconjugated AZ13599185 and AZ13687308. Additional stability experiments confirmed that quantification of the released warhead in the presence of high concentrations of MEDI4276 was acceptable. Subsequently, the assays were employed in support of a first-in-human clinical trial (NCT02576548).

## 1. Introduction

Antibody-drug conjugates (ADCs) are a class of biotherapeutic drugs that use antibodies targeting tumor-associated antigens as means to deliver covalently attached small-molecule toxins into cancer cells [1]. ADCs are comprised of three major components: Monoclonal antibody (mAb) for selective targeting, a cytotoxic drug (also referred to as warhead) and a linker molecule that conjugates the drug to the monoclonal antibody. Collectively, the warhead and the linker molecule are also referred to as the payload. Upon binding to target antigens expressed on cancer cells, ADCs are internalized into endosomes/lysosomes where the cytotoxic drug is released following cleavage of the linker. In ADCs having a non-cleavable linker, the payload is released by total digestion of the protein of component of the ADC. The released cytotoxic payload then binds to its molecular target (e.g., tubulin, DNA, or topoisomerase 1) resulting in cell cycle arrest and apoptosis [1].

ADCs have complex molecular structures, due to the combination of the molecular characteristics of small-molecule drugs and those of large-molecule biotherapeutics. Depending on the conjugation site/linker chemistry, ADCs can have a fixed or varying drug-to-antibody ratio (DAR). Biotransformation and varying clearance rates can lead to dynamically changing DAR patterns over the PK profile [2,3,4]. Bioanalysis of ADCs is challenging, due to their heterogeneous nature and their complex catabolism. Typically, three PK assays are employed to assess ADC exposure-response relationship with respect to their efficacy and safety. The three assays are: Total antibody (antibody-conjugated with payload plus any naked antibody), antibody-conjugated drug or total ADC (antibody-conjugated payload), and unconjugated drug (‘free’ payload/warhead deconjugated from antibody) [3,5,6,7]. Total antibody and total ADC concentrations are typically evaluated in relation to efficacy and on-target toxicity, while unconjugated drug concentrations are monitored for potential off-target toxicity concerns.

Ligand binding assays (LBAs) have historically been the most popular platform for large molecule bioanalysis, including quantification of total antibody and total ADC, while liquid chromatography coupled with tandem mass spectrometry (LC-MS/MS) has been the preferred platform for small molecule bioanalysis, including measurements of unconjugated payload [3,7,8]. Recently, bioanalytical scientists have been increasingly adopting hybrid immunoaffinity coupled to LC-MS/MS methods as an alternative to LBAs for large molecule bioanalysis, including total antibody and total ADC assays. In hybrid LBA-LC-MS/MS assays, the ADC molecule is isolated from the biological matrix using different capture reagents, such as protein A or G beads, anti-human Fc antibodies, anti-idiotypic antibodies, or using the therapeutic target antigen. The type or choice of capture reagent used depends on the analyte, species matrix, required detection limits and availability of reagents [7]. Following the immunocapture, the surrogate analyte (i.e., the payload/warhead (in the conjugated drug assay) or the signature peptide (in the total antibody assay)) is released using enzymatic or chemical treatment and detected using mass spectrometry [3,5,7]. It is important to note that traditional total ADC LBA assays typically measure the conjugated antibody concentration, and therefore are not DAR sensitive. In contrast, herein we describe two DAR-sensitive LBA-LC-MS/MS for quantifying total ADC from MEDI4276 and MEDI1498.

MEDI4276, an ADC developed by MedImmune LLC, is a biparatopic ADC which targets two non-overlapping epitopes on the human epidermal growth factor receptor 2 (HER-2) [9]. HER-2 is a receptor with tyrosine kinase activity which is upregulated in many cancer cells. Excessive expression of HER-2 often leads to constitutive receptor activation and therefore aggressive tumor growth [10]. Many biotherapeutic monoclonal antibodies targeting HER-2 have been approved by the United States Food and Drug Administration (FDA), including trastuzumab and pertuzumab. One of the first FDA-approved ADCs (trastuzumab emtansine) also targets HER-2. MEDI4276 was constructed by attaching the single-chain variable fragment (scFv) of trastuzumab to the amino terminus of the heavy chain of the anti-HER-2 monoclonal antibody known as 39S. 39S is a fully human IgG1 monoclonal antibody which binds to a HER-2 epitope distinct from that of trastuzumab [9]. Additionally, the biparatopic antibody contains three site mutations in the Fc regions, L234F, S239C and S442C. The two cysteine residues introduced per each heavy chain (S239C and S442C) are employed for the site-specific conjugation of the payload via a peptide-based maleimidocaproyl cleavable linker, resulting in an ADC with an average DAR of four [9]. The L234F point mutation, in combination with the S239C mutation, reduces binding to the Fc-gamma receptor (FcγR) [11], thus reducing antibody directed cytotoxicity (ADCC) effector function. MEDI4276 can crosslink HER-2 to form a large meshwork structure, due to its tetravalent binding capacity. The receptor clustering mediated by MEDI4276 enables rapid internalization of the ADC, enhanced lysosomal degradation and effective cancer cell death even in tumors with a low expression of HER-2 [11].

**Figure 1:**
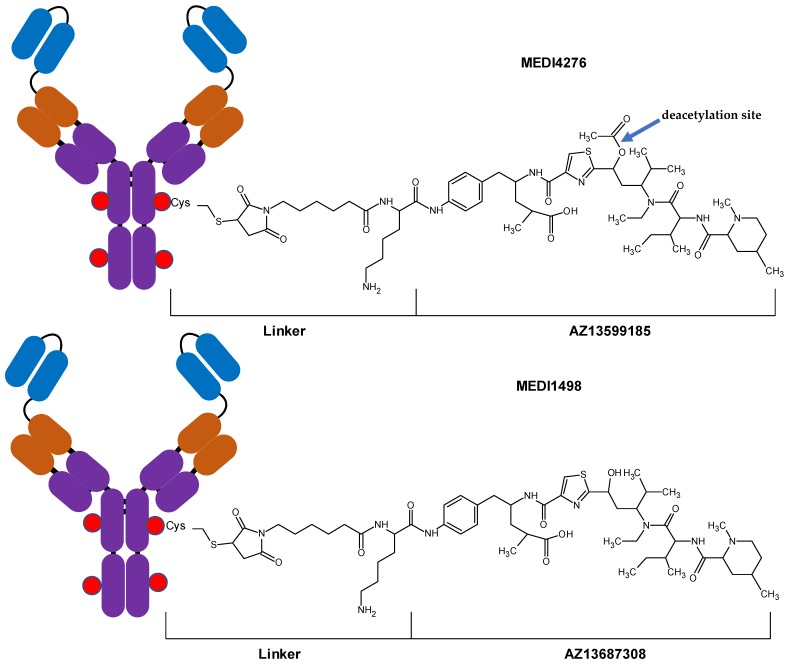
Diagrams of antibody-drug conjugates MEDI4276 and MEDI1498.

MEDI4276-conjugated warhead, AZ13599185 (AZ9185), is a variant of tubulysin with low picomolar potency. Tubulysins are antimitotic peptides originally isolated from myxobacteria [12]. Tubulysins are cytostatic/cytotoxic agents that inhibit microtubule polymerization during mitosis, resulting in cell cycle arrest, and in case of proliferating cells—cell death [12]. AZ13599185 has an ester moiety which is susceptible to cleavage resulting in deacetylation and formation of AZ13687308 (Figure 1). Loss of the acetyl moiety in tubulysins has been reported to result in significant loss in their potency [2,13,14,15]. Deacetylation of AZ13599185 can occur either while conjugated to the antibody, resulting in the deacetylated form of MEDI4276 (designated as MEDI1498) or after it has been released from the antibody. Hence, it is essential to monitor the catabolism of MEDI4276 by measuring the concentrations of MEDI1498, AZ13599185 and AZ13687308 (AZ7308) during PK assessment of MEDI4276 in clinical samples.

Therefore, PK assessment of MEDI4276 required three sophisticated bioanalytical assays: (1) Total antibody and MEDI4276 total ADC (conjugated AZ13599185), (2) MEDI1498 total ADC (conjugated AZ13687308), and (3) unconjugated AZ13599185 and unconjugated AZ13687308. Since appropriate anti-tubulysin ligand-binding reagents that could discriminate between AZ13599185 and AZ13687308 were not available, detection of the conjugated or released warhead was efficiently achieved by multiplex LC-MS/MS methods. The peptide based maleimidocaproyl linker in MEDI4276 and MEDI1498 is susceptible to enzymatic cleavage by trypsin. Digestion of MEDI4276 or MEDI1498 with trypsin resulted in the formation of smaller peptides, which are used as surrogate analytes for measuring total antibody. The digestion was accompanied by the release of the cytotoxic drug, which was used as a surrogate analyte for measuring total ADC. In this report, we describe a multiplex hybrid LBA-LC-MS/MS bioanalytical method for the measurement of total antibody and MEDI4276 total ADC in human plasma. In this method, MEDI4276 was immunocaptured using an anti-idiotypic antibody and digested with trypsin. Following proteolysis, the liberated AZ13599185 toxin was used as a surrogate analyte for the measurement of total ADC concentrations and a characteristic tryptic peptide from the complementarity-determining region (CDR) region of MEDI4276 was used as a surrogate analyte for the measurement of the total antibody concentrations. The methodology of simultaneous measurement of total ADC and total antibody has been previously proposed and demonstrated in early-stage drug development [3,16,17,18]. However, this is the first report of a validated method for the simultaneous quantification of total antibody and total ADC from an ADC therapeutic applied to a clinical trial. A second hybrid LC-MS/MS method was developed and validated for the measurement of MEDI1498 total ADC (conjugated AZ13687308) in human plasma using a nearly identical method design as was used for the MEDI4276 total ADC assay. Thus, additionally, this is the first report of a validated method for quantification of a total ADC metabolite in human plasma. A third LC-MS/MS method was developed for simultaneous measurement of unconjugated AZ13599185 and unconjugated AZ13687308 in human plasma. Currently, there are no published reports of validated bioanalytical methods to measure tubulysin-based ADCs from human matrix. Additionally, this is the first report of a validated method for the quantification of a tubulysin analogue AZ13599185 and its metabolite in human plasma.

## 2. Materials and Methods

### 2.1. Reference Materials

Antibody-drug conjugates MEDI4276 (M.W. 208,315 Da, DAR 4) and MEDI1498(M.W. 208,144, DAR 4) were supplied by MedImmune LLC, Mountain View, CA, USA. Cytotoxic tubulysin analogues AZ13599185 (M.W. 771.0 Da) and AZ13687308 (Deacetylated AZ13599185) (M.W. 729.0 Da) along with their stable isotope labeled internal standards (SIL-IS) AZ13599185-d_10_ ((2*S*,4*R*)-4-(2-((1*R*,3*R*)-1-acetoxy-3-((2*S*,3*S*)-2-((2*R*,4*R*)-l,4-dimethylpiperidine-2-carboxamido)-N-ethyl-3-[^2^H_10_] methylpentanamido)-4-methylpentyl) thiazole-4-carboxamido)-5-(4-aminophenyl)-2-methylpentanoic acid), and AZ13687308-d_10_ ((2*S*,4*R*)-5-(4-aminophenyl)-4-(2-((1*R*,3*R*)-3-((2*S*,3*S*)-2-((2*R*,4*R*)-1,4-dimethylpiperidine-2-carboxamido)-N-ethyl-3-[^2^H_10_] methylpentanamido)-1-hydroxy-4-ethylpentyl)thiazole-4-carboxamido)-2-methylpentanoic acid) were obtained from Almac (Craigavon, UK). Signature peptides SSQSVFFR (abbreviated as SSQS), C*LEWVAR (abbreviated as CLEW) and their synthetic IS peptides (abbreviated as SSQS-IS and CLEW-IS) with stable isotope labeled (^13^C_6_, ^15^N_4_) arginine (R*), SISC[CME]K-SSQSVFFR*-SNNK and APGK-C[CME]LEWVAR*IYPT (4 flanking amino acids) were purchased from Elim Biopharmaceuticals, Hayward, CA, USA.

### 2.2. Reagents

MEDI4276 anti-idiotypic antibody (Clone H2S-M9) was supplied by MedImmune, Mountain View, CA, USA. MEDI4276 anti-idiotypic antibody was biotinylated in-house using biotinylation kit (EZ-Link sulfo-NHS-LC-Biotin) purchased from Thermo Fischer Scientific, Waltham, MA, USA. Dipotassium EDTA (K2EDTA) human plasma was purchased from Biochemed, Winchester, VA, USA. High grade water was obtained using an in-house Milli-Q integral water purification system. Bovine serum albumin (BSA), dithiothreitol-DL (DTT), dimethyl sulfoxide (DMSO), ethanol, formic acid, iodoacetic acid, and isopropanol were obtained from Sigma, St. Louis, MO, USA. Acetonitrile, Methanol, and Hydrochloric Acid (6 N) were purchased from VWR Scientific, Allison Park, PA, USA. RapiGest SF Surfactant was purchased from Waters, Milford, MA, USA. Tris-buffered saline (TBS) and TBS-Tween-20 were obtained from Boston Bioproducts, Ashland, MA, USA. Ammonium bicarbonate, ammonium hydroxide (~28–0%, *v*/*v*), Dynabeads ^®^ M-280 streptavidin, MS grade trypsin protease and sodium hydroxide (NaOH) (1N) were purchased from Thermo Fisher Scientific, Waltham, MA, USA. Dubecco’s phosphate buffered saline (PBS) was purchased from Mediatech, Manassas, VA, USA. Tris-buffered saline (TBS) purchased from Boston Bioproducts, Ashland, MA, USA was used as washing buffer. Loading buffer was prepared in-house comprising of TBS-Tween-20 (Boston Bioproducts, Ashland, MA, USA) and 0.1% (*w*/*v*) bovine serum albumin.

### 2.3. Preparation of Calibration Standards and Quality Control Samples

#### 2.3.1. Assay 1: MEDI4276 Total ADC (Conjugated AZ13599185) and Total Antibody Assay

Calibration standards were prepared in K2EDTA human plasma, at a nominal total ADC (MEDI4276) concentrations of 0.025, 0.040, 0.075, 0.200, 0.600, 1.75, 4.00 and 5.00 μg/mL. The total antibody concentrations were calculated using the molecular weight of naked MEDI4276 antibody ((M.W. 203, 948 Da) and the molecular weight of ADC (M.W. 208,315 Da). The corresponding total antibody concentrations were 0.045, 0.0392, 0.0734, 0.196, 0.587, 1.71, 3.92, and 4.90 µg/mL. Calibration standard samples were freshly prepared for all validation studies. Quality control pools were prepared in K2EDTA human plasma, at nominal total ADC concentrations of MEDI4276 of 0.025, 0.060, 0.350, 3.75 and 5.00 μg/mL. Since total antibody is derived from the same reference material of MEDI4276, this corresponds to total antibody concentrations of 0.0245, 0.0587, 0.343, 3.67 and 4.90 µg/mL. The quality control samples were stored at −70 °C and −20 °C in protein low binding polypropylene tubes.

#### 2.3.2. Assay 2: MEDI1498 Total ADC (Conjugated AZ13687308) Assay

Calibration standards were prepared in K2EDTA human plasma, at a nominal MEDI1498 total ADC concentrations of 0.0250, 0.0500, 0.0750, 0.250, 1.00, 3.00, 8.00, and 10.0 μg/mL. Calibration standard samples were freshly prepared for all validation studies. Quality control pools were prepared in K2EDTA human plasma, at a nominal MEDI1498 total ADC concentrations of 0 0.0250, 0.0600, 0.500 and 7.50 μg/mL. The quality control samples were stored at −70 °C and −20 °C in protein low binding polypropylene tubes.

#### 2.3.3. Assay 3: Unconjugated AZ13599185 and AZ708 Assay

Calibration standards were prepared in K2EDTA human plasma, at nominal unconjugated AZ13599185 and AZ13687308 concentrations of 0.0500, 0.0750, 0.125, 0.300, 0.750, 2.00, 4.00 and 5.00 ng/mL. Calibration standard samples were freshly prepared for all validation studies. Quality control pools were prepared in K2EDTA human plasma, at nominal AZ13599185 and AZ13687308 concentrations of 0.0500, 0.100, 0.200, 0.500, 1.30, and 3.75 ng/mL. The quality control samples were stored at −70 °C and −20 °C in protein low binding polypropylene tubes.

### 2.4. Sample Preparation

#### 2.4.1. Assay 1: MEDI4276 Total ADC (Conjugated AZ13599185) and Total Antibody Assay

The biotinylated anti-MEDI4276 antibody was immobilized on streptavidin coated magnetic beads by incubating 40 µL of beads with 25 µL of 100 ng/mL biotinylated anti-MEDI4276 antibody for 45 min. The beads were washed and blocked with 3%, *w*/*v*, BSA in PBS. A 96-position, flat-bottom, microtiter plate was blocked with 3% BSA in PBS. A 25 µL of sample aliquot, 80 µL of loading buffer and 25 µL magnetic beads with immobilized anti-MEDI4276 antibody were added to each well. The plate was incubated overnight at 2–8 °C with a constant vortex of 750 rpm. The beads were washed four times with washing buffer (TBS, 1X) using a plate washer. Reduction and alkylation were carried out by adding 1 mg/mL RapiGest solution, 0.1 M DTT and 20:10:70 1.0 M ammonium bicarbonate/methanol/water, *v/v/v*, to each well and incubating the plate at 60 °C. Alkylation was carried out by adding 0.1 M iodoacetic acid to each well and incubating in the dark for 30 min. A 20 µL of 150 ng/mL SSQVFFR peptide internal standard (SSQS-IS) and 1600 ng/mL C[Carboxymethylcysteine(Cme)] LEWVAR peptide internal standard (CLEW-IS) working solution was added to each well. A 10 µL aliquot of 0.500 mg/mL trypsin in 50 mM ammonium bicarbonate was to each well. The plate was incubated at 37 °C in for 2 h with a constant vortexing at 700 rpm. The digestion was terminated by adding 15 µL of 2 N hydrochloric acid. A 25-µL aliquot of 3 ng/mL of AZ13599185-d_10_ working internal standard solution was added post-digestion. The digests were filtered using a 0.45 µm Millipore Multiscreen HTS Filter Plate and transferred to a 96-well, 1.0-mL deep, round-well, round-bottom, protein low binding plate. The final extracts were stored in the autosampler at 2 to 8 °C.

#### 2.4.2. Assay 2: MEDI1498 Total ADC (Conjugated AZ13687308) Assay

The sample preparation procedure was the same as the MEDI4276 total ADC and total antibody assay except for a 20 µL sample aliquot and a 20 µL aliquot of 50 ng/mL AZ13687308-d_10_ working internal standard. The peptide working internal standard was not added in this assay.

#### 2.4.3. Assay 3: Unconjugated AZ13599185 and AZ708 Assay

A 50-µL sample aliquot and 50 µL of 1.20 ng/mL AZ13599185-d_10_/AZ13599185-d_10_ internal standard working solution was added to a 96-position, 2.0-mL, conical-bottom, polypropylene plate. The plate was vortexed for 2 min at 1200 rpm and centrifuged at 1000 rpm for 1 min. 50 µL of the supernatant was transferred to a Waters Ostro phospholipid removal plate. The Waters Ostro plate was preloaded with 200 µL of 1:2 methanol/ acetonitrile, v/v, and mounted on a 1.0-mL, round-well low binding polypropylene plate. After vortexing the plate for 10 min at 1200 rpm, the mixture is eluted using 96-well positive pressure manifold. The eluate in each well was diluted with 250 µL of water prior to analysis.

### 2.5. LC-MS/MS Instrumentation

#### 2.5.1. Assay 1: MEDI4276 Total ADC (Conjugated AZ13599185) and Total Antibody Assay

High performance liquid chromatography (HPLC) system comprised of two Agilent 1200 SL binary pumps (Agilent, Palo Alto, CA, USA) and a CTC Analytics LCPAL (CTC Analytics AG, Zwingen, Switzerland) autosampler. Chromatographic separation was achieved on a Waters Acquity BEH C18, 2.1 mmx 50 mm, 1.7 µm column using 5 mM ammonium bicarbonate as mobile phase A and acetonitrile as mobile phase B. The analytical column was maintained at 50 °C. The analytical pump gradient conditions were as follows: 0.0–0.25 min, isocratic 10% B; 0.25–4.00 min, linear from 10% to 22% B; 4.00–4.50 min, linear from 22% to 90% B; 4.50–4.70 min, linear from 90% to 95%; 4.70–7.60 min, isocratic 95% B; 7.60–7.70 min, linear from 95% to 10% B; 7.70–9.00 min, isocratic 10% B. The flow rate was 0.300 mL/ min except for 5.60–7.60 mins when it was increased to 0.450 mL/min. A CTC Analytics LCPAL autosampler was used. The autosampler temperature was 2–8 °C. The injection volume was 25 µL.

Mass spectrometric detection was carried out using an AB Sciex API 5000^TM^ triple quadrupole mass spectrometer (AB Sciex, Framingham, MA, USA) with positive electrospray ionization in multiple reaction monitoring (MRM) mode (see Table 1 and Table 2 for mass spectrometric parameters). The data were acquired using Analyst Version 1.6.2 (AB Sciex, Framingham, MA, USA).

#### 2.5.2. Assay 2: MEDI1498 Total ADC (Conjugated AZ13687308)

In this assay, the HPLC system consisted of a Binary Solvent Manager and an Acquity UPLC I-Class Autosampler (Waters, Milford, MA, USA). Acquity UPLC BEH C18 VanGuard, 2.1 mm × 5 mm, 1.7 µm and Acquity BEH C18, 2.1 mm × 50 mm, 1.7 µm column (Waters, Milford, MA, USA) were used as a trapping column and an analytical column, respectively. Both trapping and analytical pumps used 10 mM Ammonium bicarbonate as mobile phase A and acetonitrile as mobile phase B. The analytical column and the trapping column were maintained at 60 °C and 65 °C, respectively.

Trapping column pump gradient conditions were as follows: 0.0–0.50 min, isocratic 10% B; 0.50–1.90 min, linear from 10% to 40% B; 1.90–2.00 min, linear from 40% to 90% B; 2.00–3.70 min, isocratic 90% B; 3.70–3.80 min, linear from 90% to 10% B; 3.80–4.50 min, isocratic 10% B. The trapping column pump was operated at a constant flow rate of 0.350 mL/min.

Analytical column pump gradient conditions were as follows: 0.0–1.50 min, isocratic 40% B; 1.50–1.51 min, linear from 40% to 90% B; 1.51–3.95 min, isocratic 90% B; 3.95–4.00 min, linear from 90% to 40% B; 4.00–4.50 min, isocratic 40% B. The analytical column flow rate was 0.150 mL/ min except for 3.60–3.95 mins when it was increased to 0.350 mL/min.

The eluate from the trapping column was sent to waste for the first 1.6 mins using a 6-port Valco switching valve. The trapping column was connected to the analytical column from 1.60–3.00 mins. After 3.00 mins, the trapping column flow was switched back to waste. The flow to the mass spectrometer was diverted from the make-up pumps to the analytical pumps from 1.60–3.00 min using a second 6-port Valco switching valve. The make-up pump had an isocratic mobile phase of 10%, *v*/*v*, acetonitrile in water being pumped at a flow rate of 0.100 mL/min. The autosampler temperature was maintained at 2–8 °C. The injection volume was 20 µL.

Mass spectrometric detection was carried out using Xevo TQ-S triple quadrupole mass spectrometer (Waters, San Jose, CA, USA) with positive electrospray ionization in MRM mode. The MRM transitions, Capillary and Cone Voltages for all the compounds are listed in Table 3. Additional mass spectrometric parameters were as follows: Source temperature was 150 °C, the desolvation temperature was 600 °C, the source offset was 70 V, the desolvation gas flow was 1000 L/h, the collision gas flow was 0.15 mL/min and the collision gas was argon. The data were acquired using MassLynx version 4.1 (Waters, San Jose, CA, USA).

#### 2.5.3. Assay 3: Unconjugated AZ13599185 and AZ708 Assay

The instrument parameters were same as Assay 2 (see Section 2.5.2) with a few modifications as described here. The HPLC system comprised of two Agilent 1200 SL binary pumps (Agilent, Palo Alto, CA, USA) and a CTC Analytics LCPAL (CTC Analytics AG, Zwingen, Switzerland) autosampler. Analytical column pump gradient conditions were as follows: 0.0–2.50 min, isocratic 40% B; 2.50–2.60 min, linear from 40% to 90% B; 2.60–4.50 min, isocratic 90% B. The analytical column flow rate was 0.150 mL/ min except for 3.60–4.50 mins when it was increased to 0.350 mL/min. The injection volume was 50 µL.

Mass spectrometric detection was carried out using ABI SCIEX API 5000 triple quadrupole mass spectrometer with positive electrospray ionization in MRM mode (see Table 1 and Table 2 for mass spectrometric parameters).

### 2.6. Method Validation Studies

Validation was carried out using guidelines set forth in the May 2001 US FDA Guidance for Industry—*Bioanalytical Method Validation* [19]. Due to the lack of an internal standard that would control the immunoaffinity isolation step, traditional LBA acceptance criteria (20/25%) were used for hybrid LC-MS/MS Assays 1 and 2 measuring total antibody and total ADC from MEDI4276 and total ADC from MEDI1498, respectively [6]. For the unconjugated AZ13599185 and AZ13687308 assay (Assay 3), traditional small molecule acceptance criteria (15/20%) were applied.

#### 2.6.1. Linearity and LLOQ

Freshly prepared calibration standards were extracted in duplicate and analyzed in 4 independent runs for each of the three assays. Assay 1, the total antibody calibration range was 0.0245 to 4.90 µg/mL (on antibody basis) and fitted using a linear regression with 1/concentration^2^ weighting, and the total ADC (conjugated AZ13599185) calibration range was 0.0250 to 5.00 µg/mL (ADC basis) and fitted using a quadratic regression with 1/concentration^2^ weighting. Assay 2, the total ADC (conjugated AZ13687308) calibration range was 0.0250 to 10.0 µg/mL (ADC basis) and fitted using a linear regression with 1/concentration^2^ weighting. Assay 3, the unconjugated AZ13599185 and AZ13687308 calibration range was 0.0500 to 5.00 ng/mL (ADC basis) and fitted using a linear regression with 1/concentration^2^ weighting. For each assay, the ratio of the peak area response of the analyte and that of the internal standard versus concentration was used. Additionally, for each assay/analyte the simplest regression that provided a balanced distribution of relative error across the curve range was selected.

#### 2.6.2. Accuracy and Precision

For Assay 1, total antibody, accuracy and precision were evaluated using quality controls samples spiked at five levels, 0.0245, 0.0587, 0.343, 3.67, and 4.90 µg/mL (antibody basis) with total ADC (conjugated AZ13599185) concentrations of 0.0250, 0.0600, 0.350, 3.75, and 5.00 µg/mL (ADC basis). For Assay 2, total ADC (conjugated AZ13687308), accuracy and precision were evaluated using quality controls samples spiked at five levels 0.0250, 0.0600, 0.500, 7.50, and 10.0 µg/mL (ADC basis). For Assay 3, unconjugated AZ13599185 and AZ13687308, accuracy and precision were evaluated using quality controls samples spiked at seven levels 0.0500, 0.100, 0.200, 0.500, 1.30, 3.75, and 5.00 ng/mL. Intra- and Inter-assay was assessed in four independent runs, with each quality control (QC) analyzed *N* = 6, processed on two different days and by at least two analysts. For Assays 1 and 2, the intra- and inter-assay precision was deemed acceptable if the coefficient of variation of the replicate determinations was less than or equal to 25.0% at the lower limit of quantification (LLOQ) level, and less than or equal to 20.0% for all other levels. The intra- and inter-assay accuracy was deemed acceptable if mean percent deviation from normal of the replicate determinations was ±25.0% at LLOQ level, and ±20.0% for all other levels. For Assay 3, the intra- and inter-assay precision was deemed acceptable if the coefficient of variation of the replicate determinations was less than or equal to 20.0% at LLOQ level, and less than or equal to 15.0% for all other levels. The intra- and inter-assay accuracy was deemed acceptable if mean percent deviation from normal of the replicate determinations was ±20.0% at LLOQ level, and ±15.0% for all other levels.

#### 2.6.3. Selectivity

Selectivity was assessed for all three assays by analyzing human plasma samples from six different sources. Selectivity requirements were that any peak area co-eluting at the retention time of analytes must be less than 20% of the peak area of the average of lowest calibration standard samples for all six lots of blank serum samples. Additionally, any peak area co-eluting at the retention time of internal standards must be less than 5% of the average internal standard peak area. In addition, these individual lots, spiked with the analytes at LLOQ, were analyzed in triplicate to evaluate fortified specificity. Fortified specificity was deemed acceptable for Assays 1 and 2 if two of the three replicates of each lot quantitated within ±25.0% of the theoretical value in at least 80% of the evaluated lots. Fortified specificity was deemed acceptable for Assay 3 if two of the three replicates of each lot quantitated within ±20.0% of the theoretical value in at least 80% of the evaluated lots.

#### 2.6.4. Cross-Analyte Interference

For each of the three assays, aliquots of blank human plasma were fortified with only one analyte at the upper limit of quantification (ULOQ) level or one internal standard at the level of use and analyzed in triplicate. Cross-analyte interference was deemed acceptable if the contribution to the response of the analyte from an interfering peak was less than 20% of the mean peak response for that analyte at LLOQ. In addition, the contribution to the response of the internal standard from an analyte sample at ULOQ level was required to be less than 5% of the mean peak response for that internal standard for that run.

#### 2.6.5. Dilutional Linearity

Dilutional linearity was assessed by diluting an over-the-curve quality control sample with blank human plasma matrix and analyzing in replicate (*n* = 6). Moreover, the procedure for diluting samples of insufficient sample volume was evaluated by diluting a mid-level quality control sample with blank matrix and analyzed in replicate (*n* = 6). The volume of the quality control sample used in this dilution was less than aliquot volume stated in the procedure for each assay. Dilutional linearity was deemed acceptable if the replicate determinations met the precision and accuracy acceptance criteria stated for the individual assay (Assays 1 and 2 (20/25%) and Assay 3 (15/20%).

#### 2.6.6. Hemolysis and Lipemia

The effect of hemolysis on the quantification was evaluated by analyzing blanks, with and without internal standard, and low- and high-level QCs, spiked with hemolyzed human whole blood to represent 5%, *v/v*, hemolysis. Similarly, the effect of lipemia on the quantification was evaluated by analyzing blanks, with and without internal standard, and low- and high-level QCs, spiked in lipemic human plasma with a triglyceride concentration of >300 mg/dL. This evaluation was deemed acceptable if the replicate determinations met the precision and accuracy acceptance criteria stated for the individual assay.

#### 2.6.7. Reinjection Reproducibility

To determine if an analytical run can be re-injected, reinjection reproducibility was evaluated by re-injecting calibration standards (CAL) and run acceptance quality controls that were stored in an autosampler. Reinjection reproducibility was deemed acceptable if the calibration standards and quality control samples met the run acceptance criteria.

#### 2.6.8. Stability Studies

Stability studies were assessed at low- and high-quality control concentrations. For post-preparative stability, the quality control samples from a precision and accuracy, stored at 2–8 °C, were reinjected with a freshly prepared calibration standard samples. Alternatively, the reinjected quality control sample responses were compared to its original calibration standard curve from the precision and accuracy run. Analyte in frozen matrix storage stability was assessed at −20 °C and −70 °C. In addition, thawed matrix stability and freeze-thaw stability was assessed. For Assay 3, unconjugated AZ13599185 and AZ13687308, frozen matrix storage stability, freeze thaw stability and thawed matrix stability were assessed in the presence of 25.0 µg/mL of MEDI4276.

#### 2.6.9. Matrix Factor

Six matrix lots (four individual plasma lots, one hemolyzed plasma lot and one lipemic plasma lot) were fortified post-extraction to approximate low and high-quality control concentration and analyzed along with a comparable external standard prepared in reagent blank. The analyte responses of the fortified matrix samples were divided by the response of the external standard (free from matrix components). The matrix factor results were also calculated using peak response ratios (IS-normalized). The matrix factor results were deemed acceptable if the coefficient of variation of the matrix factor in the six lots was less than 15% at each level.

#### 2.6.10. Recovery

##### MEDI4276 Total ADC (Conjugated AZ13599185) Assay, Total Antibody Assay, MEDI1498 Total ADC (Conjugated AZ13687308) Assay

*Immunoaffinity Capture Recovery*. The apparent recovery associated with the immunoaffinity capture was evaluated at low, medium, and high QC concentrations. Blank matrix samples, designated *pre-extraction*, were fortified with appropriate concentrations of the ADC and processed through the method as usual. A parallel set of unfortified blank matrix samples, designated *post-extraction*, were processed through the immunoaffinity capture method steps, and the same concentrations of the ADC were added to the samples just prior to the digestion (and associated pretreatment steps) and the samples were processed as usual.

*Digestion Efficiency.* The apparent recovery associated with the hydrolysis was evaluated using the above *post-extraction* samples at low, medium, and high QC concentrations. A parallel set of unfortified blank matrix samples, designated post-digestion, were processed through the immunoaffinity capture and hydrolysis steps of the method, and the same concentrations of free or AZ13599185 or AZ13687308, representing 100% recovery and hydrolysis efficiency, and internal standard were added to the samples.

##### Unconjugated AZ13599185 and AZ708 Assay

Recovery was evaluated by comparing pre-extraction and post-extraction samples at low, medium and high QC concentrations. Blank matrix samples, designated pre-extraction, were fortified with appropriate concentrations of the AZ13687308 or AZ13599185 and processed through the method as usual. A parallel set of unfortified blank matrix samples, designated post-extraction, were extracted using the method as usual. These were fortified with corresponding concentrations of AZ13687308/AZ13599185 along with internal standard post extraction.

#### 2.6.11. Carryover

The potential for carryover from a sample containing a high concentration of analyte to the following sample in an injection sequence was evaluated by injecting duplicate extracted matrix blanks immediately after the ULOQ calibration standards in each evaluation run. In addition, all samples were evaluated for carryover impact largely as described [20]. Carryover impact for two consecutively injected samples A and B was calculated using the equation carryover contribution = CM × (analyte response A/analyte response B) × 100, where CM (Carryover Multiplier) = analyte response CB1/analyte response ULOQ; CB1 = first matrix blank extract after highest-level calibration standard; Unknown A = first of two consecutively injected samples and Unknown B = second of two consecutively injected samples.

Additional experimental information can be found in the Supplemental Information. Eluting pump program for Assay 1 (Table S1A), Make-up pump program for Assay 1 (Table S1B), Valco valve program (Table S1C) for Assay 1, Eluting pump program (Table S2A) for Assay 2, Trapping pump program for Assay 2 and Assay 3 (Table S2B), Make-up pump program for Assay 2 (Table S2C), Valco valve 1 program for Assay 2 (Table S2D), Valco valve 2 program for Assay 2 (Table S2D), Valco valve program for Assay 3 (Table S2D), Eluting pump program for Assay 3 (Table S3A).

## 3. Results and Discussion

MEDI4276 consists of the tubulysin payload conjugated to a biparatopic anti-HER-2 antibody. The antibody-toxin conjugation was achieved using peptide based maleimidocaproyl linker which is susceptible to proteolytic cleavage by trypsin. Typically, pharmacokinetic assessment of antibody-drug conjugates requires three assays: Total antibody, total ADC (MEDI4276) and unconjugated toxin (AZ13599185). However, the warhead AZ13599185 has an ester moiety that is susceptible to in-vivo hydrolysis by plasma and intracellular esterases. The deacetylation has been shown to reduce the potency of tubulysin [13,21]. Hence, pharmacokinetic assessment of MEDI4276 disposition required monitoring of two additional analytes, i.e., total deacetylated ADC (MEDI1498) and unconjugated deacetylated AZ13599185 (AZ13687308). Three bioanalytical assays were developed for PK assessment, i.e., a hybrid LC-MS/MS assay for total antibody and MEDI4276 total ADC (conjugated AZ13599185), a hybrid LC-MS/MS assay for MEDI1498 total (conjugated AZ13687308), and an LC-MS/MS assay for unconjugated AZ13599185 and unconjugated AZ13687308.

### 3.1. Assay 1: MEDI4276 Total ADC (Conjugated AZ13599185) and Total Antibody Assay

**Figure 2:**
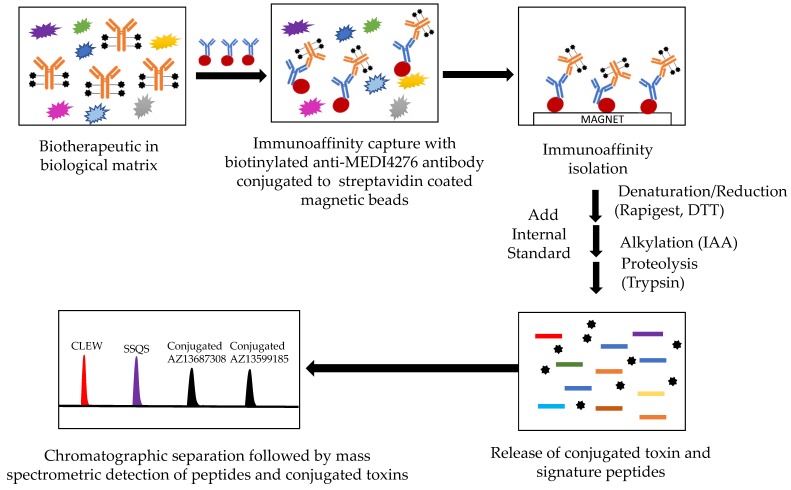
Hybrid LBA-LC-MS/MS design for simultaneous measurement of total antibody and conjugated payload (total ADC). CLEW = CLEWVAR; SSQS = SSQSVFFR.

Quantification of the total antibody or antibody-conjugated payload from an antibody-drug conjugate in a human biological matrix was achieved using a hybrid LC-MS/MS assay (See Figure 2). After immunoaffinity isolation, the captured biotherapeutic was denatured, reduced and proteolyzed with trypsin. Two peptides from the tryptic digest containing a portion of the CDR were identified and used as signature peptides for the total antibody assay. Peptide SSQVFFR (abbreviated as SSQV) from the antibody light chain was used as the quantitative peptide and C[Carboxymethylcysteine(Cme)] LEWVAR (abbreviated as CLEW) from the antibody heavy chain was used as the qualitative peptide in the total antibody. The released conjugated toxin (ac-AZ13599185) was used as the surrogate analyte for the total ADC.

Due to unavailability of a stable isotope labeled for the analyte ADC, extended stable isotope labeled (SIL) signature peptides were used as internal standards in the total antibody assessment. Extended SIL-IS peptides are added prior to digestion and can compensate for variability arising during digestion or instrumental analysis [22]. However, they do not account for variability during immunocapture. The extended peptides were synthesized containing at least four amino acids residues from the biotherapeutic protein sequence at both the N- and C- terminus. As these IS peptides were added after the reduction and alkylation steps, the peptides were synthesized with carboxymethylcysteine residues. For the total ADC quantification, a stable isotope labeled AZ13599185 was added post digestion. 

#### 3.1.1. Validation results

**Figure 3:**
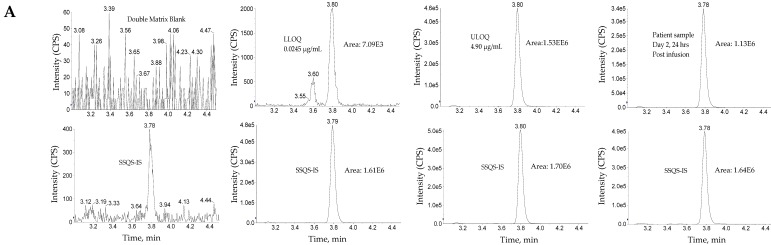

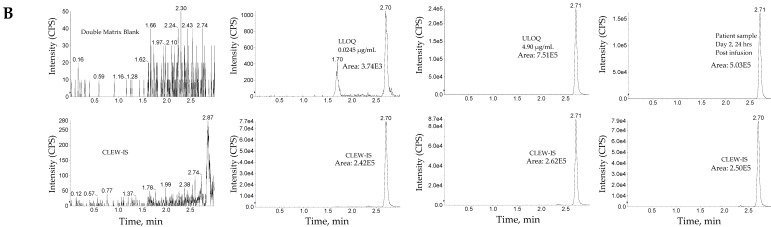

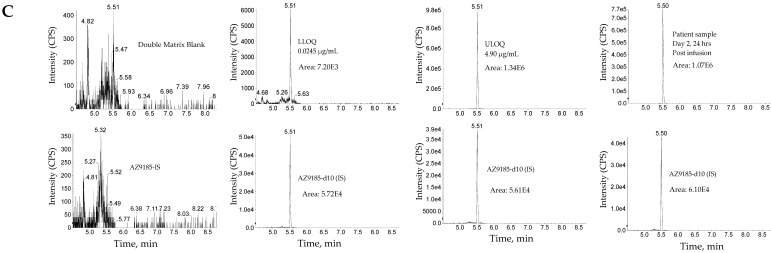
Representative chromatograms. (**A**) Total antibody assay using peptide SSQS (SSQSVFFR) as surrogate analyte. (**B**) Total antibody assay using peptide CLEW (CLEWVAR) as surrogate analyte. (**C**) Total ADC MEDI4276 using ac-AZ13599185 (AZ9185) as surrogate analyte. Peak areas were quantified using Analyst IntelliQuan processing algorithm with a smoothing factor of 3.

**Figure 4:**
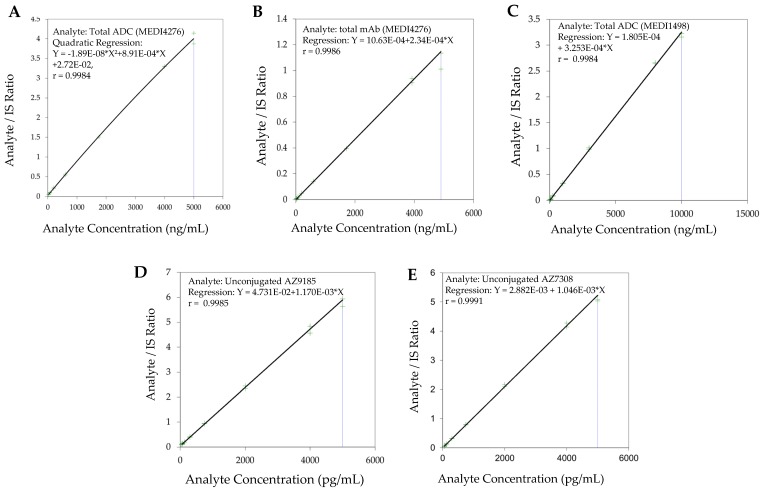
Representative calibration standard curves (**A**). Total ADC (MEDI4276) (**B**). Total antibody (MEDI4276) (**C**). Total ADC (MEDI1498) (**D**). Unconjugated AZ13599185 (AZ9185) (**E**). Unconjugated AZ13687308 (AZ7308).

The use of wider acceptance criteria (20/25%) for hybrid LC-MS/MS assays is justified especially in assays lacking an internal standard during immunoaffinity capture. The lower limit of quantification (LLOQ) was the lowest non-zero concentration level that was quantified with acceptable accuracy and precision. For this validation, the lower limit of quantification was nominally 0.0245 µg/mL for total antibody and 0.0250 µg/mL for total ADC (See Figure 3 for representative chromatograms). Method linearity was assessed over the nominal concentration range of 0.0245 µg/mL to 4.90 µg/mL for total antibody, and 0.0250 to 90 µg/mL for total ADC (See Figure 4 for representative calibration standard curves). A linear, 1/concentration^2^ weighted, least-squares regression algorithm was used to plot the peak area ratio of total antibody to its internal standard versus concentration while a quadratic, 1/concentration^2^ weighted, least-squares regression algorithm was used to plot the peak area ratio of total ADC to its internal standard versus concentration. The quadratic behavior of the curve was attributed to response saturation in the mass spectrometric detector, due to the high signal response of the conjugated payload (the molar concentration of the conjugated payload was twice the molar concentration of the antibody as MEDI4276 has a DAR of 4).

Accuracy and precision were evaluated by analyzing quality control pools prepared at 0.0245, 0.0587, 0.343, 3.67, and 4.90 µg/mL for total antibody and 0.0250, 0.0600, 0.350, 3.75, and 5.00 µg/mL for total ADC (see Table 4). Intra- and inter-assay accuracy and precision (A&P) were evaluated for each quality control pool by multiple analyses (*n* = 6) of the pool in four validation runs extract by at least two analysts on two separate days. Except for the ULOQ quality control sample (total antibody-drug conjugates, +30.6% from theoretical) in one run, the validation intra-assay data met the specified performance criteria for intra- and inter-assay accuracy and precision.

Blank matrix evaluation in six different human plasma lots did not show any significant interfering peaks at retention time of analyte or IS for total antibody and total ADC. Fortified specificity evaluation and matrix factor evaluation (% CV < 8% for both analytes) showed that matrix differences had no impact on the assay performance. In addition, the validation results showed that the hemolyzed or lipemic plasma did not hinder the assay performance (See Table 5).

Immunocapture efficiency was 178%, 189%, 154% for total antibody and 161%, 168%, 148% for total ADC at low, mid and high concentrations, respectively. The digestion efficiency was 26.7%, 27.6% and 46.9% for total antibody 16.4%, 16.9% and 29.4% for total ADC at low, mid and high concentrations, respectively. Both immunocapture efficiency and digestion efficiency results were impacted by non-specific binding occurring in the spiking solutions used for the preparation of post-immunoaffinity fortified samples, as well as post-digestion samples. During recovery evaluation, the primary criterion is to have consistent recovery across different concentrations. Consistent recovery is evident in this method from the high precision and accuracy observed for all levels in this validation.

The ability to analyze samples with insufficient volume for a full aliquot was validated by analyzing six replicate QCs, containing 0.343 µg/mL for total antibody (0.350 µg/mL for total ADC), as four-fold dilutions. The ability to dilute samples originally above the upper limit of the calibration range was validated by analyzing six replicate QCs, containing 19.6 µg/mL for total antibody (20.0 µg/mL for total ADC), as ten-fold dilutions (See Table 5). The intra-assay quality control data for the diluted QC pools was within acceptance criteria (See Table 5).

#### 3.1.2. Stability

Stability studies are summarized in Table 5. The samples were found to be stable for seven freeze/thaw cycles when stored at −70 °C or −20 °C and thawed on ice. Thawed matrix stability assessment showed that the samples were stable on the benchtop for 24 h at room temperature and on ice. Post-preparative extract stability (ES) was evaluated by comparing quality controls that were extracted and stored at 2 °C to 8 °C for approximately 87.17 h prior to reanalysis against freshly prepared calibrators. The post-preparative extract stability data met the acceptance criteria. Analyte stability in frozen matrix was evaluated by comparing stored quality control samples to freshly prepared calibration standards. The stability assessment results showed that the analyte in frozen matrix was stable for 769 days at −20 °C and −70 °C.

### 3.2. Assay 2: MEDI1498 Total ADC (Conjugated AZ13687308) Assay

Antibody-drug conjugate MEDI1498 has the same antibody and linker molecule as MEDI4276. The only difference between the two ADCs is the warhead. In MEDI1498, AZ13599185 is replaced with its deacetylated form (AZ13687308). The assay design used for measuring total ADC of MEDI1498 was same as the hybrid LC-MS/MS assay for MEDI4276. As the antibody in the MEDI1498 is same as MEDI4276, the biotinylated anti-MEDI4276 idiotypic antibody was used as the capture reagent. After washing, the captured proteins are denatured using RapiGest solution, reduced using DTT, alkylated using iodoacetic acid, and digested with trypsin. The conjugated toxin was used as the surrogate analyte in the total ADC. The freed conjugated AZ13687308 was measured as the surrogate analyte for the measurement of total ADC (MEDI1498). AZ13687308-d_10_ was used as the working standard in this assay. In comparison to the MEDI4276 total antibody and total ADC assay, the sample aliquot volume was reduced from 25 µL to 20 µL, as well as the injection volume was reduced from 25 µL to 20 µL to avoid response signal saturation at the detector.

#### 3.2.1. Validation Results

**Figure 5:**
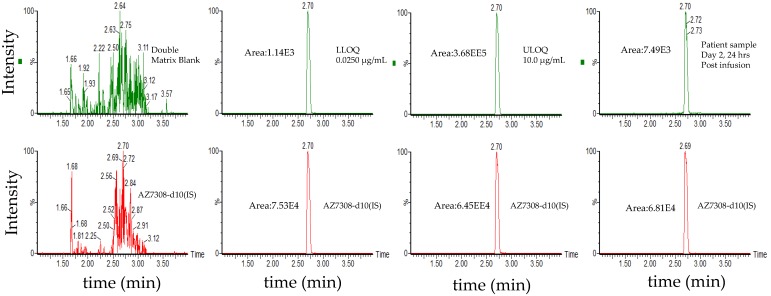
Representative chromatograms of total ADC (MEDI1498) using ac-AZ13687308 (AZ7308) as a surrogate analyte. Peak areas were quantified using Masslynx processing algorithm with a smoothing factor of 3.

The method was linear over the concertation range of 0.0250 to 10.0 µg/mL with an LLOQ of 0.250 µg/mL (See Figure 5 for representative chromatograms). A linear, least-squares regression with a weighting factor of 1/concentration^2^ was used to plot the peak area ratio of the appropriate analyte to its internal standard versus concentration (See Figure 4 for representative calibration standard curves). The average correlation coefficient from nine standard curves was >0.9900. Precision and accuracy were evaluated by analyzing quality control pools prepared at 0.0250, 0.0600, 0.500, 7.50, and 10.0 µg/mL. Intra- and inter-assay accuracy and precision (A&P) were evaluated for each quality control pool by multiple analyses (*n* = 6) of the pool in four validation runs extracted by at least two analysts on two separate days. Both intra- and inter-assay accuracy and precision were within acceptance criteria (see Table 6).

Blank matrix evaluation in six different human plasma lots did not show any interfering peaks at retention time of analyte or IS for total ADC from MEDI1498. Fortified specificity evaluation and matrix factor evaluation (%CV < 8%) showed that matrix differences had no impact on the assay performance. The back calculated concentration of quality control samples prepared with hemolyzed or lipemic plasma were within acceptance criteria (See Table 5). The ability to analyze samples with insufficient volume for a full aliquot was validated by analyzing six replicate QCs, containing 0.500 µg/mL total ADC from MEDI1498 as two-fold dilutions with blank matrix (See Table 5). Dilutional linearity was established by analyzing six replicate QCs, containing 25.0 µg/mL total ADC from MEDI1498 after ten-fold dilution with blank matrix (See Table 5). Immunoaffinity capture efficiency of the analyte from human plasma was evaluated by comparing the analyte peak responses of pre-extraction fortified (MEDI1498) samples to those of post-immunoaffinity capture fortified (MEDI1498) samples (fortified just prior to the digestion step and thus representing 100% recovery). Digestion efficiency of the analyte from human plasma was evaluated by comparing the analyte peak responses of post-immunoaffinity capture fortified (MEDI1498) samples to those of post-digestion samples (AZ13687308 fortified just prior to the digestion step and representing 100% recovery). Immunocapture efficiency was 301%, 255%, 166% at low, mid and high concentrations, respectively. The digestion efficiency was 44.9%, 49.0% and 82.5% at low, mid and high concentrations, respectively. Both immunocapture efficiency and digestion efficiency results were impacted by non-specific binding occurring in the spiking solutions used for the preparation of post-immunoaffinity fortified samples.

#### 3.2.2. Stability

Stability studies are summarized in Table 5. The samples were found to be stable for seven freeze/thaw cycles when stored at −70 °C or −20 °C and thawed on ice. Thawed matrix stability assessment showed that the samples were stable on the benchtop for 23 h on ice. Post-preparative extract stability samples were stable when stored at 2 °C to 8 °C for approximately 317.08 h. Analyte stability in frozen matrix was evaluated by comparing stored quality control samples to freshly prepared calibration standards. Analyte in frozen matrix, evaluated after 433 days at −70 °C and 97 days at −20 °C, was found to be within acceptance criteria.

##### Stability Assessment in the Presence of 25.0 µg/mL MEDI4276

Since MEDI4276 can form deacetylated MEDI1498 in vivo or in vitro, additional quality controls were prepared by fortifying 25.0 µg/mL MEDI4276 in blanks, and low- and high-level quality controls and used for stability assessment. One set of the interference quality control samples was analyzed were never frozen and analyzed fresh as nominal time zero samples. Since the MEDI4276 reference material may contain pre-existing deacetylated MEDI1498, the mean deacetylated MEDI1498 concentrations of these time-zero samples were used as the theoretical concentrations when the quality controls were used to assess additional stability studies. A mean total ADC (MEDI1498) concentration of 0.515 µg/mL was detected in blanks samples fortified with 25.0 µg/mL of MEDI4276. The mean ADC (MEDI1498) concentrations in the time-zero low- and high-quality control samples were 0.604 and 7.88 µg/mL, respectively.

The stability assessment of these MEDI4276 fortified samples met acceptance criteria for seven freeze thaw cycles at −70 °C and 24 h of thawed matrix stability on ice (%DFN ± 6.10%, %CV < 3.95%). These MEDI4276 fortified quality control samples did not meet the acceptance for post preparative extract stability when stored quality control samples were reinjected against a freshly prepared curve after 195.1 h of storage. Comparison of post preparative samples to a freshly extracted calibration standard curve approach may not always be successful, due to run-to-run analyte/IS ratio variability. Therefore, the reinjected sample extracts were quantified with their original “fresh” calibration curve with which they were analyzed on the day of extraction. The reinjected quality control sample met acceptance criteria when their responses were compared to the original calibration standard curve responses, hence indicating that there is no change in its response ratio after 195.1 h of storage (%DFN ± 2.91%, %CV < 10.9%). The MEDI4276 fortified quality control samples storage stability was acceptable after 13 days of storage at −70 °C (%DFN ± 9.50%, %CV < 6.42%).

### 3.3. Assay 3: Unconjugated Toxin and Deacetylated Toxin

Unconjugated payload analysis requires careful sample preparation techniques to minimize ADC deconjugation during sample extraction. In addition, an ester group on AZ13599185 can undergo in-vivo and ex-vivo hydrolysis to yield AZ13687308 (deacetylated AZ13599185). Hence, it is essential to measure both AZ13599185 and its metabolite AZ13687308 to evaluate the total unconjugated payload present in the clinical samples. An LC-MS/MS method was developed and validated to measure both AZ13599185 and AZ13687308 from human plasma.

A protein precipitation with acetonitrile was used for extraction of the analytes from plasma. Protein precipitated extracts are commonly susceptible to matrix effects, due to the presence of high amounts of co-eluting lipids which can result in ion suppression or enhancement. In order to reduce such matrix effects, sample extracts can be depleted of phospholipids using commercially available phospholipid removal plates. In this method, after protein precipitation, the samples were centrifuged and the supernatant was passed through a Waters OstroTM phospholipid removal plate to reduce matrix effects.

**Figure 6:**
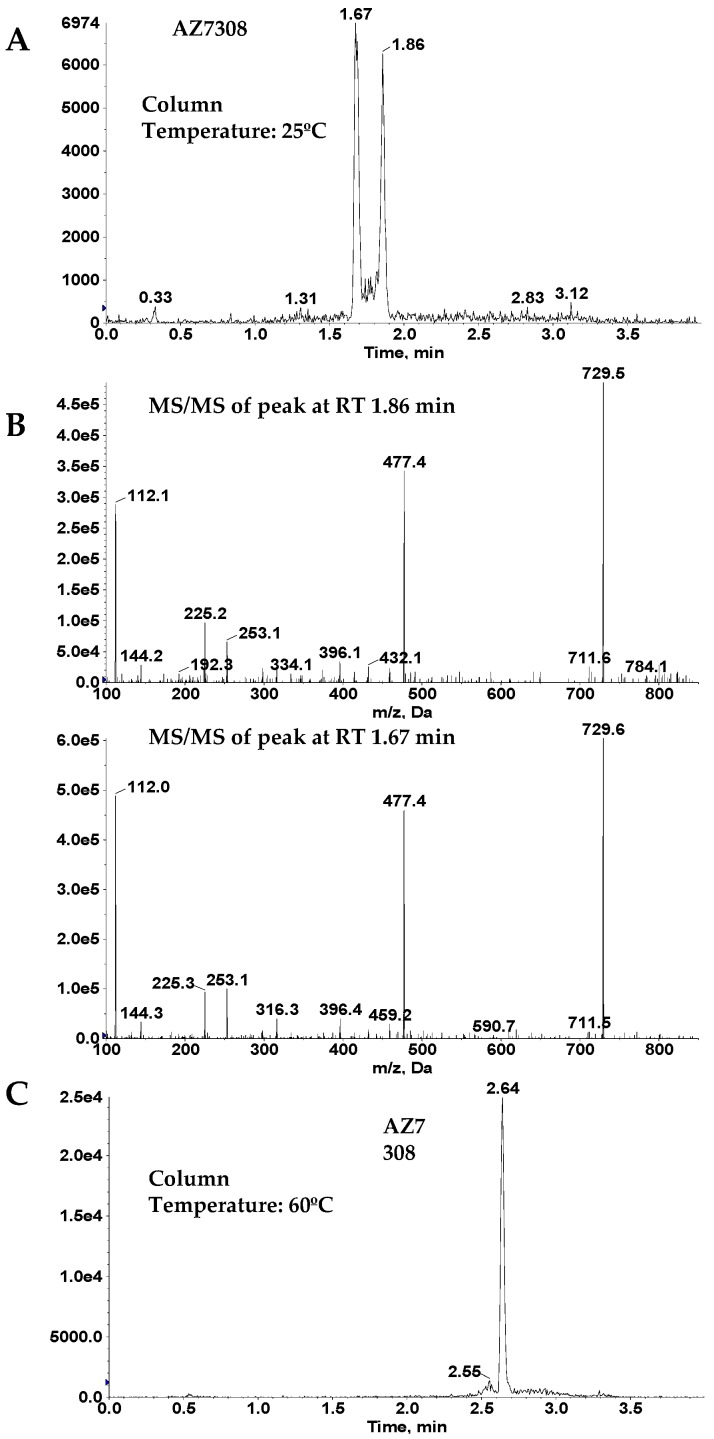
Effect of column temperature on AZ13687308 (AZ7308) peak shape. (**A**) Peak split observed at 25 °C. (**B**) Mass spectrometric fragmentation spectra of the two peaks observed in (**A**). (**C**) Single peak observed at 60 °C.

In addition, due to a relatively high chromatographic background of the protein extracts, online column trapping technique was utilized for sample enrichment prior to separation on the analytical column. The sample was loaded onto the trapping column using a using a binary gradient consisting of 10 mM ammonium bicarbonate and acetonitrile. The initial eluate from the trapping column containing salts and hydrophilic analytes were directed to waste. After 2.4 min, the trapping column was connected to the analytical column. By increasing the organic composition of the gradient, the analytes were eluted from the trapping column and separated on the analytical column prior to mass spectrometric detection. A split peak was observed for analyte AZ13687308 during initial chromatographic evaluation, however, this was not observed for AZ13599185. Fragmentation pattern confirmed that both peaks were AZ13687308, indicating isomeric peak distortion. When the column temperature was raised, the peak shape merged into a single peak (See Figure 6). This phenomenon can probably be attributed to the loss of shape selectivity at elevated temperatures on C18 columns [23]. This phenomenon was not observed for AZ13599185 probably due to the relatively bulky acetyl moiety in comparison to the free hydroxy moiety in AZ13687308. In the optimized method, the column temperature was maintained at 60 °C.

**Figure 7:**
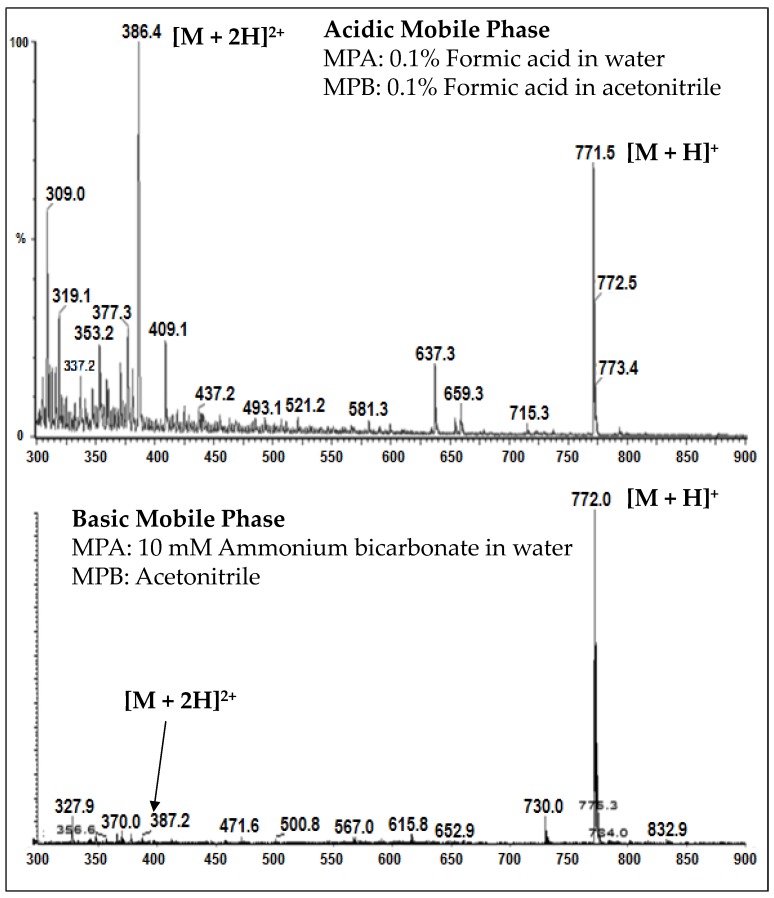
Charge-state distribution of AZ13599185 in acidic and basic mobile phases.

A charge distribution of AZ13599185 and AZ13687308 precursor ions was observed wherein the analytes ionized to form both [M+H]+ and [M+2H]2+ ions at similar abundance under acidic conditions. When ionization was carried out using basic mobile phases, the two charged states coalesced into a single [M+H]+ charge state (See Figure 7). Hence, the optimized method employs a basic mobile phase system to achieve better sensitivity through charge state coalescence enabling lower detection limits. 

#### 3.3.1. Validation results

**Figure 8:**
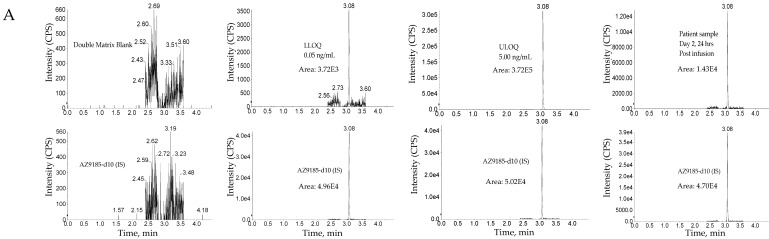

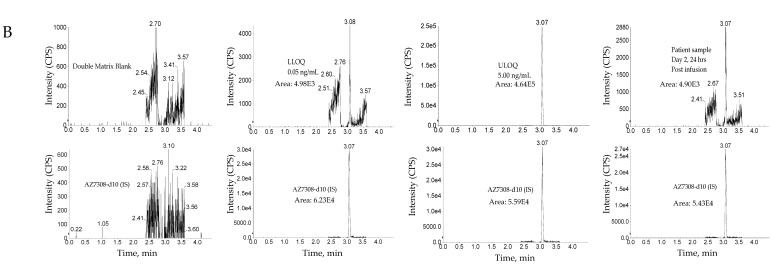
Representative chromatograms. (**A**) Unconjugated AZ13599185 (AZ9185) assay. (**B**) Unconjugated AZ13687308 (AZ7308) assay. Peak areas were quantified using Analyst IntelliQuan processing algorithm with a smoothing factor of 3.

The method was linear over the concertation range of 0.0500 to 5.00 ng/mL with an LLOQ of 50 pg/mL for AZ13599185 and AZ13687308 (See Figure 8 for representative chromatograms). A linear, 1/concentration^2^ weighted, least-squares regression algorithm was used to plot the peak area ratio of the appropriate analyte to its internal standard versus concentration (See Figure 4 for representative calibration standard curves). The average correlation coefficient from nine standard curves was > 0.9900 for both analytes. Accuracy and precision were evaluated by analyzing quality control pools prepared at 0.0500, 0.100,0.200,1.30,3.75 and 5.00 ng/mL for AZ13599185 and AZ13687308. Intra- and inter-assay accuracy and precision (A&P) were evaluated for each quality control pool by multiple analyses (*n* = 6) of the pool in at least four validation runs extracted by at least two analysts on two separate days. Both intra- and inter-assay accuracy and precision were within acceptance criteria (see Table 7). 

Blank matrix evaluation in six different human plasma lots did not show any interfering peaks at retention time of analyte or IS for AZ13599185 and AZ13687308. Fortified specificity evaluation and matrix factor evaluation (%CV < 5% for both analytes) showed that matrix differences had no impact on assay performance. The back calculated concentration of quality control samples prepared with hemolyzed or lipemic plasma were within acceptance criteria (See Table 5). Extraction recovery was 76.5%, 74.5%, 69.8% for AZ13599185 and 75.0%, 74.1%, 71.7% for AZ13687308 at low, mid and high concentrations, respectively.

During cross analyte interference evaluation, a chromatographic peak was detected at the mass transition and expected retention time of AZ13687308 in the cross-analyte interference samples fortified with AZ13599185 that was approximately 27.0% of the mean LLOQ response in that run. The presence of AZ13687308 detected in the cross-analyte interference samples fortified with AZ13599185 is most likely due to a low-level impurity of AZ13687308 in the AZ13599185 reference standard material. The presence of this impurity in the reference standard was further supported by the direct analysis of external standards of the individual analytes prepared in dilution solution (1:2 methanol/acetonitrile *v*/*v*) at the same nominal concentration as the extracted analyte interference check samples. In this validation, the samples were fortified with an equal amount of AZ13599185 and AZ13687308 at each level. Hence, based on the extracted ULOQ standard responses of AZ13687308, the potential bias in the determination of AZ13687308 introduced from an impurity in the AZ13599185 reference standard is expected to be <0.206% at each level.

The ability to analyze samples with insufficient volume for a full aliquot was validated by analyzing six replicate QCs, containing 1.30 ng/mL AZ13599185 and AZ13687308, as two-fold dilutions (See Table 5). The ability to dilute samples originally above the upper limit of the calibration range was validated by analyzing six replicate QCs, containing 10.0 ng/mL AZ13599185 and AZ13687308, as ten-fold dilutions (See Table 5). The intra-assay quality control data for the diluted QC pools was within acceptance criteria.

#### 3.3.2. Stability

Stability studies are summarized in Table 5. The samples were stable for seven freeze/thaw cycles when stored at −70 °C or −20 °C and thawed on ice for both analytes. Thawed matrix stability assessment showed that the samples were stable on the benchtop for 24 h at room temperature and on ice. Post-preparative extract stability samples were stable when stored at 2 °C to 8 °C for approximately 55.03 h for both analytes. Analyte in frozen matrix stability assessment, evaluated after 856 days of storage at −70 °C and after 77 days of storage at −20 °C, was found to be within acceptance criteria.

##### Stability Assessment in the Presence of 100.0 µg/mL MEDI4276

Due to the expected presence of high concentrations (up to 100 µg/mL) of MEDI4276 in incurred samples, it was important to assess the potential impact on the quantification of AZ13599185 and AZ13687308. For comparison, if AZ1985 was entirely deconjugated from a solution of 100 µg/mL MEDI4276, it will result in a solution with a concentration 1500 ng/mL of AZ13599185. This concentration is three hundred-fold higher than the upper limit of quantification in this assay.

To evaluate the impact of the presence of high concentration of MEDI4276 on this assay, interference quality control samples (blank, low- and high-level quality controls) were prepared containing 100.0 µg/mL MEDI4276. One set of these interference quality control samples was analyzed were never frozen and analyzed fresh as time zero samples. Since the MEDI4276 reference material may contain pre-existing AZ13599185 and AZ13687308, the mean concentrations of these time-zero samples were used as the theoretical concentrations when these interference quality controls were used to assess additional stability studies. A mean AZ13599185 concentration of 0.0395 ng/mL was seen in blanks samples fortified with 100 µg/mL of MEDI4276. The mean AZ13599185 concentrations in the time-zero interference low- and high-quality control samples were 0.235 and 3.79 ng/mL, respectively. AZ13687308 was not detected in blanks samples fortified with 100 µg/mL of MEDI4276. The mean AZ13599185 concentrations in time-zero interference low- and high-quality control samples were 0.170 and 3.19 ng/mL, respectively.

The stability assessment of these MEDI4276 fortified samples met acceptance criteria for seven freeze thaw cycles at −70 °C (%DFN ± 3.44%, %CV < 6.97%). and 24 h of thawed matrix stability on ice (%DFN ± 4.72%, %CV < 4.29%). for both analytes. The MEDI4276 fortified quality control samples storage stability was acceptable after 21 days of storage at −70 °C for both analytes (%DFN ± 3.44%, %CV < 4.50%).

### 3.4. Carryover

The potential for carryover from a sample containing a high concentration of the analyte to the following sample in an injection sequence was evaluated by injecting duplicate extracted matrix blanks immediately after the ULOQ calibration standards in each evaluation run. There were contributions from chromatographic peaks, at the expected retention time of the analyte in the blank samples, greater than 20% of the mean analyte response for the LLOQ calibration standards in some of the validation runs for all three assays. To ensure carryover does not impact quantification, blank matrix samples were injected after samples with an expected high concentration in subsequent runs. In addition, all samples were assessed for potential impact due to carryover. Any sample having a potential impact of greater than 5% were flagged for reanalysis.

## 4. Conclusions

PK assessment of complex biotherapeutics, such as ADCs, typically requires multiple assays: Total antibody, total ADC and unconjugated payload. Additional assays are required if the analytes are known to undergo additional biotransformations. Three multiplex LC-MS/MS assays for the quantification of five distinct analytes were developed and validated for bioanalysis of antibody-drug conjugate MEDI4276 in humans. The payload AZ13599185 in MEDI4276 is known to undergo deacetylation. Hence, antibody-conjugated and unconjugated deacetylated AZ13599185 (AZ13687308) were also quantified.

The total antibody and total ADC in human plasma were measured by a multiplex hybrid LBA-LC-MS/MS assay. While the methodology used herein has been proposed and demonstrated previously [3,16,17,18], this publication highlights the first report of its utilization for bioanalysis of a total antibody and a conjugated tubulysin toxin in humans for *clinical applications*. The report highlights a hybrid approach of combining immunoaffinity and LC-MS/MS analysis, as well as a single enzymatic step to release the conjugated payload and total antibody signature peptide from ADC biotherapeutic. An anti-MEDI4276 idiotypic antibody was used as a capture reagent while proteolysis was carried out with trypsin. Two signature peptides were identified and monitored for total antibody assay. The conjugated payload was used as a surrogate analyte for the total ADC assay. The method demonstrated acceptable precision and accuracy over the range of 0.0245 to 4.90 µg/mL and 0.0250 to 5.00 µg/mL for total antibody and total ADC, respectively. Stability studies demonstrated acceptable bench-top, post-preparative, freeze-thaw and storage stability.

As has been reported with other tubulysin warheads [13], AZ13599185 is known to undergo deacetylation in-vivo while attached to the antibody and this affects the potency of MEDI4276 (unpublished report). To account for any deacetylation of the warhead in the ADC biotherapeutic, a second hybrid LC-MS/MS assay was developed and validated for the measurement of antibody-conjugated AZ13687308 (deacetylated AZ13599185). MEDI1498, a metabolite of MEDI4276 with conjugated AZ13687308, was used as the reference standard in this assay. As MEDI1498 has the same antibody and linker components as MEDI4276, the MEDI4276 total ADC assay immunocapture and digestion scheme was utilized in this assay. The validation results showed that the method was accurate and precise with good linearity over the range of 0.0250 to 10.0 µg/mL. Stability assessment was conducted with and without the presence of high levels of MEDI4276 (25.0 µg/mL). The samples showed acceptable freeze-thaw, bench-top and good bench-top, post preparative and storage stability.

Unconjugated warhead assay is an important ADC assay for exposure-safety assessment. A simple method that utilizes protein precipitation extraction and LC-MS/MS analysis was developed for the measurement of AZ13599185 and AZ13687308 in human plasma. It is important that unconjugated payload assays have low detection limits to ensure measurement of trace levels of the liberated toxin. In this method, the analyte response for AZ13599185 was enhanced by adjusting the pH of the mobile phase to allow charge coalescence during mass spectrometric ionization (Figure 7). The method demonstrated acceptable precision and accuracy for both analytes. The method was linear over the range of 0.0500–5.00 ng/mL for both analytes. Stability assessment was conducted with and without the presence of high levels of MEDI4276 (100 µg/mL) and demonstrated that the presence of the parent drug did not impact quantification of the released toxin. The samples demonstrated acceptable freeze-thaw, thawed matrix, post-preparative and storage stability.

In summary, three assays were developed, validated and deployed for the measurement of five analytes from MEDI4276 in human plasma. The analytes were total antibody, conjugated payload (total ADC), conjugated payload metabolite, unconjugated payload, and unconjugated payload metabolite. The methods were applied to PK analysis of clinical samples. PK data for each of the analytes from two representative subjects are presented in Table 8 and Table 9. Additionally, representative sample chromatograms are presented in Figure 3, Figure 5 and Figure 8. This is the first report of a multiplex hybrid LC-MS/MS assay for simultaneous measurement of total antibody and conjugated toxin from an ADC in a *human matrix*. The report also describes a sensitive method for quantification of unconjugated tubulysin analogue AZ13599185 and AZ13687308 with a low detection limit of 50 pg/mL from human plasma. This report illustrates the multiplexing capabilities of LC-MS/MS platform to enable efficient bioanalysis of an ADC along with its major metabolites for clinical applications.

## Figures and Tables

**Table 1:** Tandem mass spectrometry (MS/MS) parameters ABI SCIEX API 5000.

Analyte	tR (min)	Dwell Time (ms)	Q1 (m/z)	Q3 (m/z)	DP (V)	CE (V)	CXP (V)	EP (V)
CLEW	2.3	100	467.6	531.3	50	27	31	7
CLEW-IS	2.3	100	472.6	541.4	50	27	31	7
SSQS (Total Antibody)	3.66	150	479.4	655.4	35	23	24	4
SSQS-IS	3.66	150	484.4	665.4	35	23	24	4
ac-AZ13599185	5.4	50	771.4	519.3	50	53	19	5
ac-AZ13599185-d_10_	5.4	50	781.4	519.3	50	53	19	5
AZ13599185	3.1	50	771.5	519.3	100	44	17	10
AZ13599185-d_10_	3.1	50	781.5	519.3	100	44	17	10
AZ13687308	3.1	25	729.5	447.3	100	44	17	10
AZ13687308-d_10_	3.1	25	739.5	447.3	100	44	17	10

CE-collision energy, CXP-collision cell exit potential, EP-entrance potential, CLEW and SSQS are proteotypic peptides with the following sequences: SSQSVFFR and C[Carboxymethylcysteine(Cme)] LEWVAR.

**Table 2:** Source Parameters ABI SCIEX API 5000.

MS Parameter	Assay 1 *	Assay 3 **
Ion Source Temp (TEM):	600 °C	500 °C
IonSpray Voltage (IS):	5500 V	5500 V
Collision Gas Flow (CAD):	5	12
Curtain Gas Flow (CUR):	25	30
Nebulizer Gas Flow (NEB/GS1):	50	30
Turbo IonSpray Gas (AUX/GS2):	60	60
Processing Software	Analyst software, Version 1.6.2	

* Assay 1: MEDI4276 total antibody-drug conjugates (ADC) (Conjugated AZ13599185) and total antibody assay, ** Assay 2: MEDI1498 total ADC (Conjugated AZ13687308) assay.

**Table 3:** MS/MS Parameters for Waters Xevo TQ-S Triple Quadrupole Mass Spectrometer.

Analyte	tR (min)	Dwell Time (ms)	Q1 (m/z)	Q3 (m/z)	Cone Voltage (V)	Collision Voltage (V)
AZ13687308	2.70	100	729.6	477.3	78	36
AZ13687308-d_10_	2.70	100	739.6	477.3	78	36

**Table 4:** Total antibody (MEDI4276) and total ADC (MEDI4276) precision and accuracy.

Analyte	Total Antibody (SSQS Signature Peptide)			MEDI4276 Total ADC (ac-AZ13599185)		
Acceptance Criteria	Limits of 20% (25%) of LLOQ			Limits of 20% (25%) of LLOQ		
	Conc. (µg/mL)	Precision	Accuracy	Conc. (µg/mL)	Precision	Accuracy
QC Intra-assay Statistics (%)	0.0245	4.55 to 12.4%	−7.31% to −1.78%	0.025	3.52 to 9.04%	−5.97% to 5.12%
	0.0587	2.99 to 6.77%	−1.49% to 2.46%	0.0600	1.55 to 8.33%	−6.40% to 1.03%
	0.343	1.28 to 2.26%	−0.0456% to 6.12%	0.350	2.74 to 3.65%	−5.40% to 4.39%
	3.67	2.35 to 5.97%	−3.84% to 2.58%	3.75	2.85 to 7.15%	−1.91% to 4.84%
	4.90	2.11 to 5.59%	−0.546% to 5.31%	5.00	6.19 to 6.68%	5.32% to 30.6%
QC Inter-assay Statistics (%)	0.0245	8.65%	−4.93%	0.025	7.47%	1.55%
	0.0587	4.53%	0.70%	0.06	5.54%	−2.11%
	0.343	2.85%	2.47%	0.35	4.86%	−0.37%
	3.67	4.44%	−0.18%	3.75	5.50%	1.55%
	4.9	4.23%	2.58%	5	10.10%	17.00%

**Table 5:** Summarized validation and stability results.

Analyte (*n* = 6)	Total Antibody (MEDI 4276)		Total ADC (MEDI 4276)		Total ADC (MEDI 1498)		Unconjugated AZ13599185		Unconjugated AZ13687308	
	%DFN	%CV	%DFN	%CV	%DFN	%CV	%DFN	%CV	%DFN	%CV
Dilutional linearity	−4.47	3.79	−4.19	6.56	−7. 33	4.53	7.12	2.23	4.71	2.42
Insufficient volume	−5.87	4.55	−6.22	4.15	2.3 4	3.93	3.97	3.34	7.31	1.06
Hemolyzed Plasma	±7.20	<7.78	±14.4	<3.94	±11.1	<5.55	±10.3	<9.29	±0.916	<4.82
Lipemic Plasma	±3.43	<7.46	±2.17	<3.54	±3. 56	<5.28	±10.0	<7.06	±11.4	<3.55
Post-preparative Extract Stability (2 to 8 °C)	±5.14	<4.15	±7.29	<3.69	±11.4	<4.92	±2.83	<4.97	±5.12	<6.64
	87.17 h		87.17 h		317.08 h		55.03 h		55.03 h	
Thawed Matrix Stability (On Ice)	±0.540 *	<6.32 *	±3.22	<3.40	±8.41	<5.13	±6.04	<4.35	±5.91	<6.84
	24 h		24 h		23 h		24 h		24 h	
Freeze Thaw Stability (7 cycles, −20 °C)	±5.54	<11.0	±1.76	<4.04	±10.1	<9.26	±4.03	<6.78	±3.90	<3.46
Freeze Thaw Stability (7 cycles, −70 °C)	±2.97	<6.94	±2.47	<7.72	±4.91	<15.2	±7.16	<3.02	±10.1	<5.28
Storage stability (−20 °C)	±14.9	<4.20	±13.4	<4.38	±3.53	<7.29	±3.36	<3.50	±0.907	<2.96
	769 days		769 days		97 days		77 days		77 days	
Storage stability (−70 °C)	±12.0	<7.22	±13.0	<4.30	±14.4	<10.7	±5.89	<4.25	±3.76	<8.86
	769 days		769 days		433 days		856 days		856 days	

* Thawed matrix stability was evaluated on ice and at room temperature.

**Table 6:** Total ADC (MEDI1498) Precision and Accuracy.

Analyte	MEDI1498 Total ADC (ac-AZ13687308)		
	Conc. (µg/mL)	Precision	Accuracy
QC Intra-assay Statistics (%)	0.025	6.56 to 13.1%	−14.1 to 12.0%
	0.0600	2.83 to 8.74%	−4.30 to 10.2%
	0.500	2.04 to 4.23%	−2.58 to 3.21%
	7.50	0.920 to 8.79%	1.16 to 7.33%
	10	1.54 to 7.56%	−3.22 to 4.35%
QC Inter-assay Statistics (%)	0.025	12.50%	−1.26%
	0.0600	7.84%	1.88%
	0.500	3.52%	0.12%
	7.50	5.68%	3.89%
	10	4.30%	0.70%

**Table 7:** Unconjugated AZ13599185 and AZ13687308 precision and accuracy.

Analyte	AZ13599185			AZ13687308		
Acceptance Criteria	Limits of 15 (20 at LLOQ)			Limits of 15 (20 at LLOQ)		
	Conc. (µg/mL)	Precision	Accuracy	Conc. (µg/mL)	Precision	Accuracy
QC Intra-assay Statistics (%)	0.05	3.75 to 10.2%	−0.0990 to 15.0%	0.05	4.19 to 10.4%	1.97 to 11.4%
	0.100	3.50 to 8.21%	−3.73 to 8.42%	0.1	3.44 to 6.57%	3.49 to 9.99%
	0.200	1.98 to 8.87%	0.765 to 13.6%	0.2	2.79 to 9.62%	6.05 to 16.6%
	0.5	1.93 to 13.0%	2.31 to 11.1%	0.5	1.44 to 11.9%	4.82 to 15.5%
	1.3	1.86 to 5.89%	0.676 to 10.9%	1.3	1.90 to 5.46%	1.78 to 16.3%
	3.75	0.969 to 4.01%	3.35 to 10.3%	3.75	1.24 to 2.37%	5.33 to 18.7%
	5	1.81 to 12.2%	−4.93 to 5.88%	5	1.53 to 12.5%	2.61 to 7.16%
QC Inter-assay Statistics (%)	0.05	8.66%	7.53%	0.05	7.50%	6.23%
	0.1	7.10%	3.85%	0.1	5.29%	5.70%
	0.2	6.48%	8.91%	0.2	6.37%	9.70%
	0.5	6.89%	7.25%	0.5	6.27%	9.26%
	1.3	5.06%	6.47%	1.3	5.59%	8.59%
	3.75	3.44%	7.30%	3.75	4.50%	10.20%
	5	6.45%	2.64%	5	5.92%	5.21%

**Table 8:** Representative pharmacokinetic (PK) data. Data for total mAb, total ADC from MEDI4276 and MEDI1498 from a representative subject (ID# 20016190001).

VISIT.	Planned Time Point Variable	Total mAb (ng/mL)	Total ADC, MEDI4276 (ng/mL)	Total ADC, MEDI1498 (ng/mL)
VISIT 2 (DAY 1)	PRE-DOSE	<24.50	<25.00	<25.00
VISIT 2 (DAY 1)	END OF INFUSION	5678.02	4848.96	226.70
VISIT 2 (DAY 1)	POST- INFUSION 2 h	5044.61	4997.90	199.93
VISIT 2 (DAY 1)	POST- INFUSION 6 h	5190.82	5030.96	206.90
VISIT 3 (DAY 2)	POST- INFUSION 24 h	3981.13	3828.52	208.04
VISIT 4 (DAY 3)	POST- INFUSION 48 h	2584.60	2556.41	195.38
VISIT 5 (DAY 8)	POST- INFUSION 168 h	<24.50	<25.00	<25.00
VISIT 6 (DAY 15)	POST- INFUSION 336 h	<24.50	<25.00	<25.00

**Table 9:** Representative pharmacokinetic (PK) data. Data for AZ13599185 and AZ13687308 from a representative subject (ID# 20016160003).

VISIT	Planned Time Point Variable	AZ13599185 (pg/mL)	AZ13687308 (pg/mL)
VISIT 2 (DAY 1)	PRE-DOSE	<50.00	<50.00
VISIT 2 (DAY 1)	END OF INFUSION	<50.00	<50.00
VISIT 2 (DAY 1)	POST- INFUSION 2 h	50.78	<50.00
VISIT 2 (DAY 1)	POST- INFUSION 6 h	67.91	<50.00
VISIT 3 (DAY 2)	POST- INFUSION 24 h	195.58	52.70
VISIT 4 (DAY 3)	POST- INFUSION 48 h	279.28	<50.00
VISIT 5 (DAY 8)	POST- INFUSION 168 h	148.27	<50.00
VISIT 6 (DAY 15)	POST- INFUSION 336 h	82.78	<50.00

## Supplementary Materials

The following are available online at http://www.mdpi.com/2073-4468/8/1/11/s1, Table S1A: Eluting pump program Assay 1, Table S1B: Make-up pump program Assay 1, Table S1C: Valco valve program Assay 1, Table S2A: Eluting pump program Assay 2, Table S2B: Trapping pump program Assay 2 and Assay 3, Table S2C: Make-up pump program Assay 2, Table S2D: Valco valve 1 program Assay 2, Table S2D: Valco valve 2 program Assay 2, Table S2D: Valco valve program Assay 3, Table S3A: Eluting pump program Assay 3.
