# Supplementary material for: Multiplex LC-MS/MS Assays for Clinical Bioanalysis of MEDI4276, an Antibody-Drug Conjugate of Tubulysin Analogue Attached via Cleavable Linker to a Biparatopic Humanized Antibody against HER-2
Source: Antibodies (Basel). 2019 Jan 11;8(1):11. doi: 10.3390/antib8010011 (PMC6640689; doi:10.3390/antib8010011)
Supplement: Supplementary file 1 [file antibodies-08-00011-s001.pdf]

Supplementary

Table S1A: Eluting pump program Assay 1

Step	Total Time (min)	Flow Rate (μL/min)	Composition B (%)
0	0.00	300	10
1	0.25	300	10
2	4.00	300	22
3	4.50	300	90
4	4.70	300	95
5	5.50	300	95
6	5.60	450	95
7	7.60	450	95
8	7.70	300	10
9	9.00	300	10

Table S1B: Make-up pump program Assay 1

Step	Total Time (min)	Flow Rate (μL/min)	Composition B (%)
0	0.00	300	70
1	9.00	300	70

Table S1C: Valco valve program Assay 1

Total Time (min)	Position	Comments
Initial	A	Flow from Makeup pump to MS
1.60	B	Flow from column to MS
6.30	A	Flow from Makeup pump to MS

Table S2A: Eluting pump program Assay 2

Step	Total Time (min)	Flow Rate (μL/min)	Composition B (%)
0	0.00	150	40
1	1.50	150	40
2	1.51	150	90
3	3.50	150	90
4	3.60	350	90
5	3.95	350	90
6	4.00	150	40
7	4.50	150	40

Table S2B: Trapping pump program Assay 2 and Assay 3

Step	Total Time (min)	Flow Rate (μL/min)	Composition B (%)
0	0.00	350	10
1	0.50	350	10
2	1.90	350	40
3	2.00	350	90
4	3.70	350	90
5	3.80	350	10
6	4.50	350	10

Table S2C: Make-up pump program Assay 2

Step	Total Time (min)	Flow Rate (μL/min)	Composition B (%)
0	0.00	100	10
1	4.50	100	10

Table S2D: Valco valve 1 program Assay 2

Total Time (min)	Position	Comments
Initial	A	Flow from Makeup pump to MS
1.60	B	Flow from analytical column to MS
3.00	A	Flow from Makeup pump to MS

Table S2D: Valco valve 2 program Assay 2

Total Time (min)	Position	Comments
Initial	A	Trap column to waste
1.60	B	Transfer the analytes from trapping column to analytical column
3.00	A	Trapping column equilibrium and analytical column wash

Table S2D: Valco valve program Assay 3

Total Time (min)	Position	Comments
Initial	A	Trap column to waste
2.40	B	Transfer the analytes from trapping column to analytical column
3.60	A	Trapping column equilibrium and analytical column wash

Table S3A: Eluting pump program Assay 3

Step	Total Time (min)	Flow Rate (μL/min)	Composition B (%)
0	0.00	150	40
1	2.50	150	40
2	2.60	150	90
3	3.50	150	90
4	3.60	350	90
5	4.50	300	90
